# Innate signalling molecules as genetic adjuvants do not alter the efficacy of a DNA-based influenza A vaccine

**DOI:** 10.1371/journal.pone.0231138

**Published:** 2020-04-03

**Authors:** Dennis Lapuente, Viktoria Stab, Michael Storcksdieck genannt Bonsmann, Andre Maaske, Mario Köster, Han Xiao, Christina Ehrhardt, Matthias Tenbusch

**Affiliations:** 1 Institute of Clinical and Molecular Virology, University Hospital Erlangen, Friedrich-Alexander University Erlangen-Nürnberg, Erlangen, Germany; 2 Department of Molecular and Medical Virology, Ruhr-University Bochum, Bochum, Germany; 3 Environmental Medicine, UNIKA-T Augsburg, Technische Universität München and Helmholtz Zentrum, Neuherberg, Germany; 4 Model Systems for Infection and Immunity, Helmholtz Centre for Infection Research, Braunschweig, Germany; 5 Section of Experimental Virology, Institute of Medical Microbiology, University Hospital Jena, Jena, Germany; Stanford University School of Medicine, UNITED STATES

## Abstract

In respect to the heterogeneity among influenza A virus strains and the shortcomings of current vaccination programs, there is a huge interest in the development of alternative vaccines that provide a broader and more long-lasting protection. Gene-based approaches are considered as promising candidates for such flu vaccines. In our study, innate signalling molecules from the RIG-I and the NALP3 pathways were evaluated as genetic adjuvants in intramuscular DNA immunizations. Plasmids encoding a constitutive active form of RIG-I (cRIG-I), IPS-1, IL-1β, or IL-18 were co-administered with plasmids encoding the hemagglutinin and nucleoprotein derived from H1N1/Puerto Rico/8/1934 via electroporation in BALB/c mice. Immunogenicity was analysed in detail and efficacy was demonstrated in homologous and heterologous influenza challenge experiments. Although the biological activities of the adjuvants have been confirmed by *in vitro* reporter assays, their single or combined inclusion in the vaccine did not result in superior vaccine efficacy. With the exception of significantly increased levels of antigen-specific IgG1 after the co-administration of IL-1β, there were only minor alterations concerning the immunogenicity. Since DNA electroporation alone induced substantial inflammation at the injection site, as demonstrated in this study using Mx2-Luc reporter mice, it might override the adjuvants´ contribution to the inflammatory microenvironment and thereby minimizes the influence on the immunogenicity. Taken together, the DNA immunization was protective against subsequent challenge infections but could not be further improved by the genetic adjuvants analysed in this study.

## Introduction

Seasonal influenza infections are estimated to cause about three to five million cases of severe illness including 290,000 to 650,000 deaths [1]. Groups most at risk for severe illness are young children, pregnant women, immunocompromised people, and the elderly. Currently, trivalent and quadrivalent inactivated influenza vaccines (TIV/QIV) as well as live attenuated influenza vaccines (LAIV) are on the market as seasonal vaccines. TIV/QIV are aimed to induce neutralizing antibodies against the highly variable surface protein hemagglutinin (HA) but the efficacy is rather moderate especially in the elderly (50–60%) [2,3]. Moreover, the induced antibody responses are highly strain-specific and do not lead to protection against mismatched viruses in seasons where unpredicted strains occur [4]. In contrast, LAIV are able to induce cross-reactive T cell responses that provide some degree of immunity against heterologous influenza A Virus (IAV) strains [5], but so far, they were only effective in children and even this efficacy varied substantially in recent years [6–8].

Therefore, alternative vaccines capable of inducing cellular and humoral immune responses that provide long-lasting protection against a broader range of IAV strains are highly desired.

The induction of broadly neutralizing antibodies able to prevent infections with multiple IAV strains is highly desirable. Unfortunately, these antibody responses are only rarely induced upon natural infection or classical vaccination approaches. Thus, more sophisticated strategies like the sequential immunization with chimeric HA variants [9] or with headless HAs [10] were developed but are still under preclinical and clinical evaluation. In contrast to broadly neutralizing antibodies, cross-reactive T cell responses against conserved target proteins do not lead to a sterile immunity but can be easily induced and decrease morbidity and mortality upon an heterologous IAV infection in animal models [11–14]. Observational studies during the H1N1 pandemic in 2009 have recapitulated the cross-protective potential of T cell immunity in humans [15,16]. We and others used genetic vaccines based on viral vectors or plasmid DNA to induce T cell responses against the conserved nucleoprotein (NP) [17–22]. Although DNA immunizations showed promising results in these preclinical studies, they lacked immunogenicity in human individuals [23]. However, delivery methods like the electroporation can increase the immunogenicity of naked DNA plasmids to a level suitable for use in clinical studies [24–27].

In the present study, we hypothesized that the co-delivery of molecules from innate immune pathways might further increase the immunogenicity of a DNA vaccine. Specifically, the RIG-I (Retinoic acid inducible gene I) and the NALP3 (NACHT, LRR and PYD domains-containing protein 3) inflammasome axes were selected as targets because they are essential for the innate recognition of natural IAV infections and the instruction of adaptive immune responses (reviewed in [28]). The RIG-I pathway starts with the activation of the cytoplasmic RNA sensor RIG-I and the downstream oligomerization of its adaptor protein IPS1 (interferon-β promoter stimulator 1; also known as MAVS or VISA) leading to the production of pro-inflammatory cytokines and type I IFN responses [29–31]. The overexpression of full-length IPS1 [29] or a constitutive active variant of RIG-I (cRIG-I; [32]) is sufficient to promote these responses. Several studies have shown that RIG-I pathway induction influences the initiation of T cell responses upon viral infection [33,34] and that the efficacy of experimental IAV vaccines can be boosted by co-administration of RIG-I agonists [35–37]. The NALP3 inflammasome complex is assembled upon recognition of pathogen- or damage-associated signals. This leads to the activation of caspase-1, which in turn cleaves Interleukin-1β (IL-1β) and Interleukin-18 (IL-18) from their inactive precursors into the bioactive forms and further facilitates their secretion (reviewed in [38]). IL-1β is a highly bioactive cytokine involved in acute tissue inflammation [39]. Numerous studies have described the multifaceted functions of this cytokine in attracting innate and adaptive immune cells into inflamed tissues [40–44] as well as its direct T cell stimulatory effects [45,46]. Similarly, IL-18 was shown to mediate the attraction of T cells [47], their T_H_1/T_H_2 commitment [48], and the proliferation of cytotoxic CD8^+^ T cells [49–52].

Here, we evaluated the individual and combined use of components from the RIG-I (cRIG-I, IPS1) and NALP3 pathways (IL-1β, IL-18) as genetic adjuvants in intramuscular DNA immunizations against influenza A virus infection. Our aim was to boost the immunogenicity in order to enhance homologous as well as heterologous immunity.

## Material and methods

### DNA plasmids for immunization

Antigen-encoding DNA plasmids were constructed by insertion of codon-optimized gene sequences for hemagglutinin (HA) and nucleoprotein (NP), both derived from H1N1 A/Puerto Rico/8/1934 (PR8), into the pVax1 backbone (Invitrogen). Antigen expression is initiated from a CMV-immediate/early-1-promoter and a bovine BGH polyadenylation signal provides transcription termination. Similarly, murine DNA sequences encoding the adjuvants mature IL-1β, mature IL-18 (in both constructs a TPA leader sequence was fused N-terminally to provide efficient secretion in the absence of Caspase-1-mediated processing), cRIG-I (only encoding the CARD domain from the first 284 amino acids of RIG-I), and IPS1 (full-length) were inserted into pVax1, respectively. A pVax1 backbone without insert was used as an empty vector control (pVax-empty or pVax is most figures). The integrity of the expression cassettes was confirmed by PCR and transgene expression was detected by Western blot. Plasmid DNA was prepared with the NucleoBond Xtra Maxi EF Kit (Macherey-Nagel). Endotoxin levels were measured by QCL-1000 Chromogenic LAL assay (Lonza; all preparations <0.0001 endotoxin units/immunization dose).

### Cell culture

The human embryonic kidney cell line HEK293T (ATCC CRL-3216), the mouse melanoma cell line B16 (ATCC CRL-6323), and the Madin-Darby canine kidney cell line MDCK II (ATCC CRL-2936) were maintained in DMEM supplemented with 10% FCS and 1% penicillin/streptomycin (both Gibco) at 37°C and 5% CO_2_. The murine fibroblast reporter cell line Mx/RAGE7 [53] was maintained in DMEM supplemented with 10% FCS, 1% penicillin/streptomycin, 1 mM sodium pyruvate, and 50 μM 2-mercaptoethanol at 32°C and 5% CO_2_.

### Luciferase promoter reporter assays

Confluent B16 cells were transfected in a 96-well plate with 0.1 μg of the respective reporter plasmid for promoter (binding) activity of IFN-β, IRF3, NFκB, AP-1, STAT3, ISRE or p53, respectively, 0.2 μg of the adjuvant plasmids (or a GFP-encoding control plasmid), and 0.3 μg polyethylenimine in DMEM supplemented with 10% FCS and 1% penicillin/streptomycin. After 48 hours incubation at 37°C and 5% CO_2_, the cells were lysed with 100 μl Glo-lysis buffer, 25 μl Bright-Glo Luciferase substrate (both Promega) was added, and the signal was measured on a microplate luminometer (Orion L, Titertek Berthold).

### Type I IFN reporter cell line

For the analysis of type I IFN induction, a virus free, cell-based assay was used [53]. Briefly, the adjuvant plasmids, an IFN-α2 plasmid, or a GFP-encoding vector (as negative control) were transfected into HEK293T cells (24-well plate, 2 μg/well plasmid, 2 μg/well polyethylenimine). After 48 hours of cultivation, supernatants were cleared and 100 μl of each was given on Mx/RAGE7 reporter cells in a 96-well plate. After 24 hours at 37°C, the supernatants were omitted and 200 μl medium was added for another 48 hours. Then, cells were detached using Trypsin/EDTA (Gibco) and stained with a live/dead dye for exclusion of dead cells in the later analysis (Fixable Viability Dye eFluor® 780; 1:4000 eBioscience). The type I IFN-dependent GFP expression in the reporter cells was assessed by flow cytometry (BD FACS Canto II) using FlowJo software (Tree Star Inc.).

### Mice and immunizations

Six to eight-weeks-old female BALB/cJRj mice were purchased from Janvier (Le Genest-Saint-Isle, France) and Mx2-Luc mice were kindly provided by Mario Köster (HZI, Braunschweig, Germany). Mice were housed in individually ventilated cages in accordance with German law and institutional guidelines. The research staff was trained in animal care and handling in accordance to the FELASA and GV-SOLAS guidelines. The study was approved by an external ethics committee authorized by the North Rhine-Westphalia State Office for Consumer Protection and Food Safety (license 84–02.04.2013-A371). The duration of the experiments was between 7 and 43 days. A score sheet was used to recognize humane endpoints based on body weight changes, behaviour, and appearance. The most important criterion for a humane endpoint during the influenza infections was the weight loss; if an animal lost more than 30% of its weight relative the starting point of the infection or more than 25% of its weight and did not start to regain weight within 48h, the respective animal was euthanized immediately. None of the animals died during the infections before meeting these criteria.

Mice were immunized intramuscularly in both shaved hind legs (musculus gastrocnemius) under anaesthesia (100 mg/kg ketamine and 15 mg/kg xylazine). The indicated amounts of DNA plasmids were injected in a volume of 30 μl PBS per leg followed by local electroporation about one second later [54]. Electroporation was performed with a 4-electrode array that applied electric signals of 63 V amplitude and 40 ms total duration (Ichor Medical Inc.). Blood samples were collected from the retro-orbital sinus under anaesthesia with inhaled isoflurane.

### Analysis of *in vivo* antigen expression

Mice were immunized (as described above) with the respective adjuvant (or pVax-empty as control) plus a luciferase-encoding vector as reporter antigen. At the indicated time points, mice were anaesthetized with isoflurane and 200 μg D-Luciferin was injected in a volume of 30 μl PBS in both leg muscles. The luciferase signal was measured three minutes later with an IVIS Lumina Series II (PerkinElmer).

### FACS-based antibody analysis

A flow cytometric antibody analysis was performed to analyse antigen-specific antibody responses in sera [54]. Therefore, HEK 293T cells were transfected with plasmid DNA encoding the respective antigen of interest together with plasmids encoding a blue fluorescent protein (BFP; 5 μg BFP-encoding plasmid plus 20 μg antigen-encoding plasmid per 75 cm^2^ flask). Two days later, the cells were incubated for 20 minutes at 4°C with serum samples diluted either in FACS-PBS (PBS with 0.5% BSA and 1 mM sodium azide) to bind to HA on the surface, or in permeabilization buffer (0.5% saponin in FACS-PBS) to bind to intracellular NP. After a washing step, antigen-specific antibodies were detected with polyclonal anti-mouse Ig-FITC (1:300, 4°C, 20 min incubation; BD Biosciences). The median FITC fluorescence intensity of transfected BFP^+^ cells was measured on a BD FACSCanto II and analysed using FlowJo software (Tree Star Inc.).

### Influenza microneutralization assay

Influenza-specific neutralizing antibody responses were quantified in a microneutralization assay [55]. Therefore, two-fold serial dilutions of serum samples were prepared in infection medium (DMEM containing 0.18% BSA, 1% penicillin/streptomycin and 1.2 μg/ml Trypsin; Gibco). Those dilutions were then incubated with 2000 plaque-forming units (PFU) H1N1 A/Puerto Rico/8/1934 (PR8) or pH1N1 A/Hamburg/4/2009 (pandemic isolate from 2009; pdm09) for 45 minutes at 37°C in a total volume of 100 μl infection medium. After the incubation, the serum-virus mixtures were transferred on confluent MDCK II cells in a 96-well plate. After four days cultivation at 37°C, neutralization of the virus was assessed in each well by crystal violet staining (0.13% w/v crystal violet, 26% Methanol in water). The highest reciprocal sample dilution, which completely inhibited an infection, was considered as the neutralization titer.

### Antigen-specific antibody ELISA

Antigen-specific antibody responses were analysed by an ELISA as described in our previous study [21]. In short, ELISA plates were coated with 5x10^5^ PFU heat-inactivated influenza virus in 100 μl carbonate buffer (50 mM carbonate/bicarbonate, pH 9.6) per well over night at 4°C. After blocking free binding sites with 5% skimmed milk in PBS-T (PBS containing 0.05% Tween-20) for one hour at RT, serum dilutions in 2% skimmed milk in PBS-T were added to the wells for one hour at RT. After three washing steps each with 200 μl PBS-T, specifically bound antibodies were detected by incubation with HRP-coupled antibodies against murine IgG1 and IgG2a, respectively, for one hour at RT (all 1:1000 dilution; clone X56 and clone R19-15; BD Biosciences). Subsequently, the plates were washed seven times with PBS-T and after the addition of ECL solution, the signal was measured on a microplate luminometer (Orion L, Titertek Berthold).

### Intracellular cytokine staining

At the indicated time points, T cell responses in PBMCs or splenocytes were analysed. Spleens were mashed through a 70 μm cell strainer before ammonium-chloride-potassium lysis, while blood was directly subjected to this treatment. 10^6^ splenocytes or PBMCs were plated per well in a 96-well round-bottom plate and incubated for 6 hours at 37°C and 5% CO_2_ in 200 μl R10 medium (RPMI 1640 supplemented with 10% FCS, 2 mM l-Glutamine, 10 mM HEPES, 50 μM 2-mercaptoethanol and 1% penicillin/streptomycin) containing monensin (2 μM), anti-CD28 (1 μg/ml, eBioscience), anti-CD107a-FITC (clone eBio1D4B, eBioscience) and 5 μg/ml of the MHC-I peptides HA_518–526_ (IYSTVASSL) or NP_147–155_ (TYQRTRALV), respectively. Non-stimulated samples were used for subtraction of background cytokine production (negative values were set as zero). After the stimulation, cells were stained with anti-CD8a-Pacific blue (clone 53–6.7, BD Biosciences; 1:300) and Fixable Viability Dye eFluor® 780 (eBioscience; 1:2000) for 20 min at 4°C. After fixation in 2% formaldehyde in PBS and permeabilization (buffer and conditions see above), cells were stained intracellularly with anti-IL-2-APC (clone JES6-5H4, BD Biosciences), anti-TNFα-PECy7 (clone MPG-XT22, BD Biosciences), and anti-IFN-y-PE (clone XMG1.2, eBioscience; all 1:300) for 30 min at 4°C. Data were acquired on a BD FACSCanto II and analysed using FlowJo software (Tree Star Inc.).

### Influenza virus infections

Mice were experimentally infected with H1N1 A/Puerto Rico/8/1934 (PR8) or pH1N1 A/Hamburg/4/2009 (pdm09; infectious dose is indicated in the respective experimental part). Under anaesthesia (100 mg/kg ketamine and 15 mg/kg xylazine), mice got an intranasal instillation of the indicated infectious dose in a volume of 50 μl PBS. Weight loss and animal health was monitored daily until the end of the experiment, which was usually between six and eight days post-infection. Bronchoalveolar lavage fluid (BALF) and lung tissue were conducted with two times 1 ml PBS to analyse viral replication and cellular infiltration. The viral RNA was quantified by qRT-PCR as described elsewhere [55].

### Flow cytometric analyses of cellular infiltration

BALF from infected mice were centrifuged (5 min, 5000×g) and one quarter of the cellular fraction was stained with anti-Gr1-FITC (clone RB6-8C5, eBioscience, 1:1000), anti-CD49b-PE (clone DX5, eBioscience, 1:1000), anti-CD45-PerCP (clone 30-F11, BD Biosciences, 1:300), anti-CD19-PE-Cy7 (clone 1D3, BD Biosciences, 1:2000), anti-F4/80-APC (clone BM8, eBioscience, 1:300), anti-CD11b-APC-Cy7 (clone M1/70, BD Biosciences, 1:300), anti-CD11c-Pacific-Blue (clone HL3, BD Biosciences, 1:300), and anti-CD3e-BV510 (clone 145-2C11, BD Biosciences, 1:100) for 20 min at 4°C. CD4 and CD8 T cells were stained in a second staining panel with anti-CD8-Pacific blue (clone 53–6.7, BD Biosciences, 1:300) and anti-CD4-PerCP (clone RM4-5, eBioscience, 1:2000).

### Assessment of *in vivo* type I IFN induction

Mx2-Luc mice [56] were immunized by intramuscular DNA injection followed by electroporation as described above. 72 hours later, mice were euthanized and muscles of the hind legs were excised. The muscle tissues were homogenized with a gentleMACS dissociator in M tubes (Miltenyi Biotec) and 10 μl of the cleared supernatant was incubated with 90 μl Bright-Glo lysis buffer (Promega) for 10 minutes at RT. Subsequently, 25 μl Bright-Glo Luciferase substrate (Promega) was added and the luciferase signal was measured with an IVIS Lumina Series II (PerkinElmer).

### Statistical analyses

Results are shown as mean + SEM or as median ± interquartile range. Statistical analyses were performed with Prism 5.0 (GraphPad Software, Inc.). Non-parametric tests (Kruskal-Wallis non-parametric one-way ANOVA followed by Dunn's post-test and non-parametric two-way ANOVA followed by Dunnett's multiple comparisons) were used because normality testing for small datasets has little power. A *p* value of <0.05 was considered to be statistically significant. For reasons of clarity, only significant differences between the controls (naive, pVax-empty) and the adjuvanted groups are displayed in most figures. Raw data is available as S1 Raw data file.

## Results

### *In vitro* inflammation induced by the genetic adjuvants

**Fig 1:**
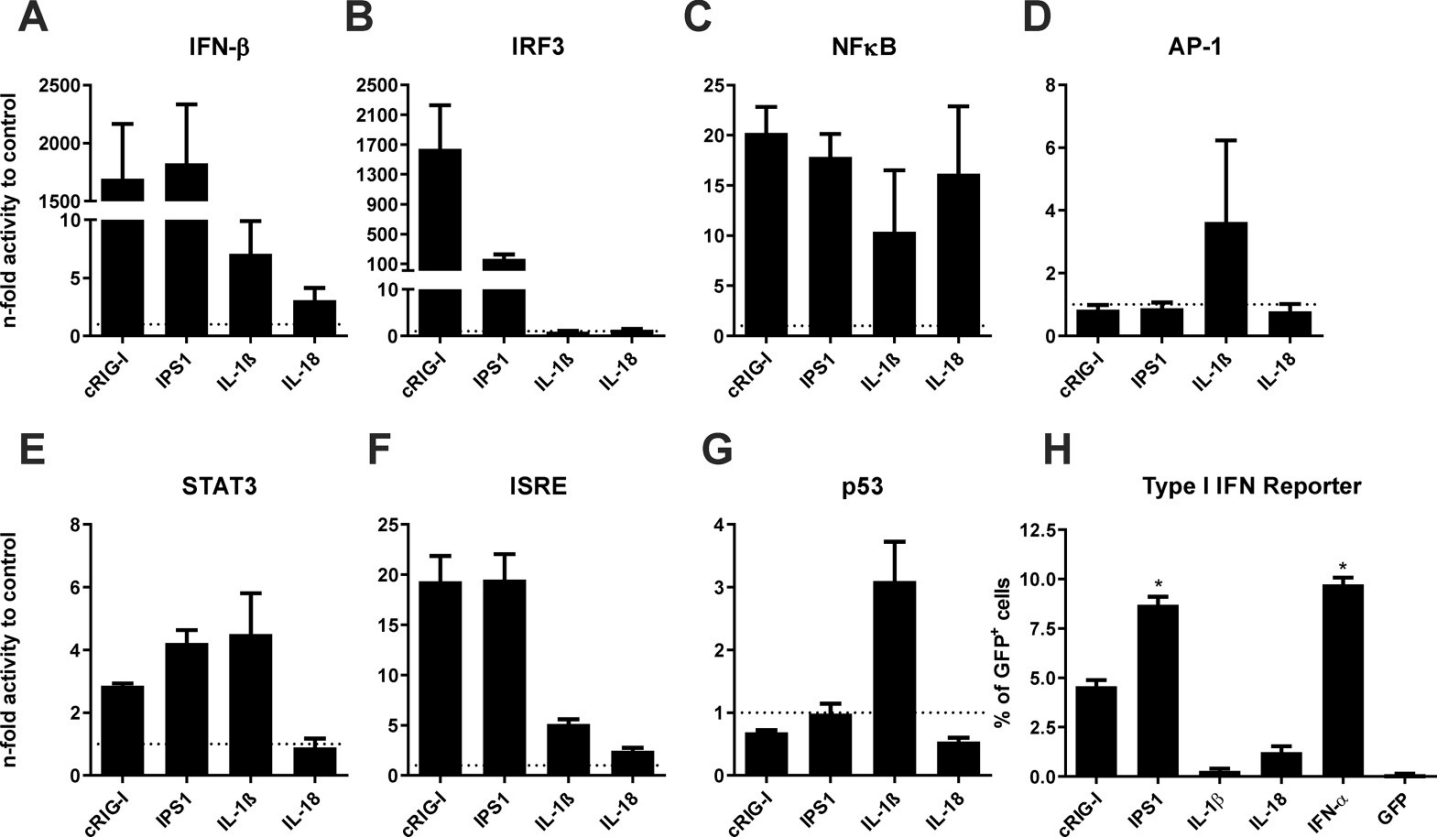
*In vitro* bioactivity of genetic adjuvants. (A-G) Murine B16 cells were co-transfected with genetic adjuvants and the respective luciferase promoter reporter construct. 48 hours later, cells were lysed and the luciferase signal measured as a surrogate for respective promoter activity. Values are expressed as n-fold promoter activity relative to the co-transfection with a GFP-encoding plasmid as control. Depicted are means + SEM of triplicates. IFN-β, Interferon-β; IRF3, interferon regulatory factor 3; NFκB, nuclear factor 'kappa-light-chain-enhancer' of activated B-cells; AP-1, activator protein 1; STAT3, Signal transducer and activator of transcription 3; ISRE, interferon-sensitive response element; p53, tumor protein 53. (H) HEK 293T cells were transfected with the genetic adjuvants, an IFN-α2-encoding plasmid as positive control, or a GFP-encoding plasmid as negative control. 48 hours later, supernatants were transferred on Mx/RAGE7 reporter cells. The type I IFN-induced GFP expression in the reporter cells was assessed by flow cytometry 72 h later. Depicted is the percentage of GFP+ cells (+ SEM) within the live cell compartment. Statistical significances were analysed by Kruskal-Wallis non-parametric one-way ANOVA followed by Dunn's post-test; *, p<0.05 vs. GFP.

First, we analysed whether the adjuvants show bioactivity in a murine B16 cell line. Therefore, the respective adjuvant plasmids pV-cRIG-I (constitutively active form of RIG-I), pV-IPS1 (interferon-beta promoter stimulator 1), pV-IL-1β (Interleukin-1β), or pV-IL-18 (Interleukin-18) were co-transfected with specific promoter reporter plasmids for inflammatory/apoptotic pathways and the luciferase signal, which correlates with promoter activity, was analysed. Normalized to the transfection with a control vector (indicated by the dotted line), transfection with pV-cRIG-I or pV-IPS1 strongly induced the type I IFN axis as indicated by the promoter activity for IRF3, IFN-β, and to some extent by the secondary activation of ISRE (Fig 1A, 1B and 1F). Moreover, the RIG-I-related adjuvants had moderate effects on NFκB and STAT3 (Fig 1C and 1E) indicating the production of pro-inflammatory cytokines as well. Regarding the NALP3 adjuvants, pV-IL-18 mainly activated the NFκB pathway but a slightly increased IFN-β and ISRE promoter activity was observed in addition (Fig 1A, 1C and 1F). The expression of IL-1β induced a broader inflammation than IL-18. Beside an activation of the NFκB, IFN-β, and ISRE pathways it initiated AP-1, STAT3, and p53 signalling (Fig 1D, 1E and 1G). Although only moderately, the p53 promoter was solely affected by IL-1β but not by the other adjuvants, perhaps indicating the induction of apoptosis/pyroptosis by IL-1β.

As a second proof of bioactivity, we used Mx/RAGE7 reporter cells, which express the green fluorescent protein (GFP) driven by the IFN-inducible Mx1 promoter [53]. Therefore, these cells can be used to detect type I IFNs in a quantitative manner. While IPS1 induced amounts comparable to the direct production from an IFN-α2-encoding plasmid, cRIG-I led to a somewhat lower type I IFN production (Fig 1H). Interestingly, cells transfected with pV-IL-18 but not pV-IL-1β produced moderate levels of type I IFNs compared to supernatants from cells transfected with a GFP-encoding plasmid as negative control. Thus, the DNA-encoded adjuvants induce specific patterns of inflammation.

### *In vivo* persistence of antigen

**Fig 2:**
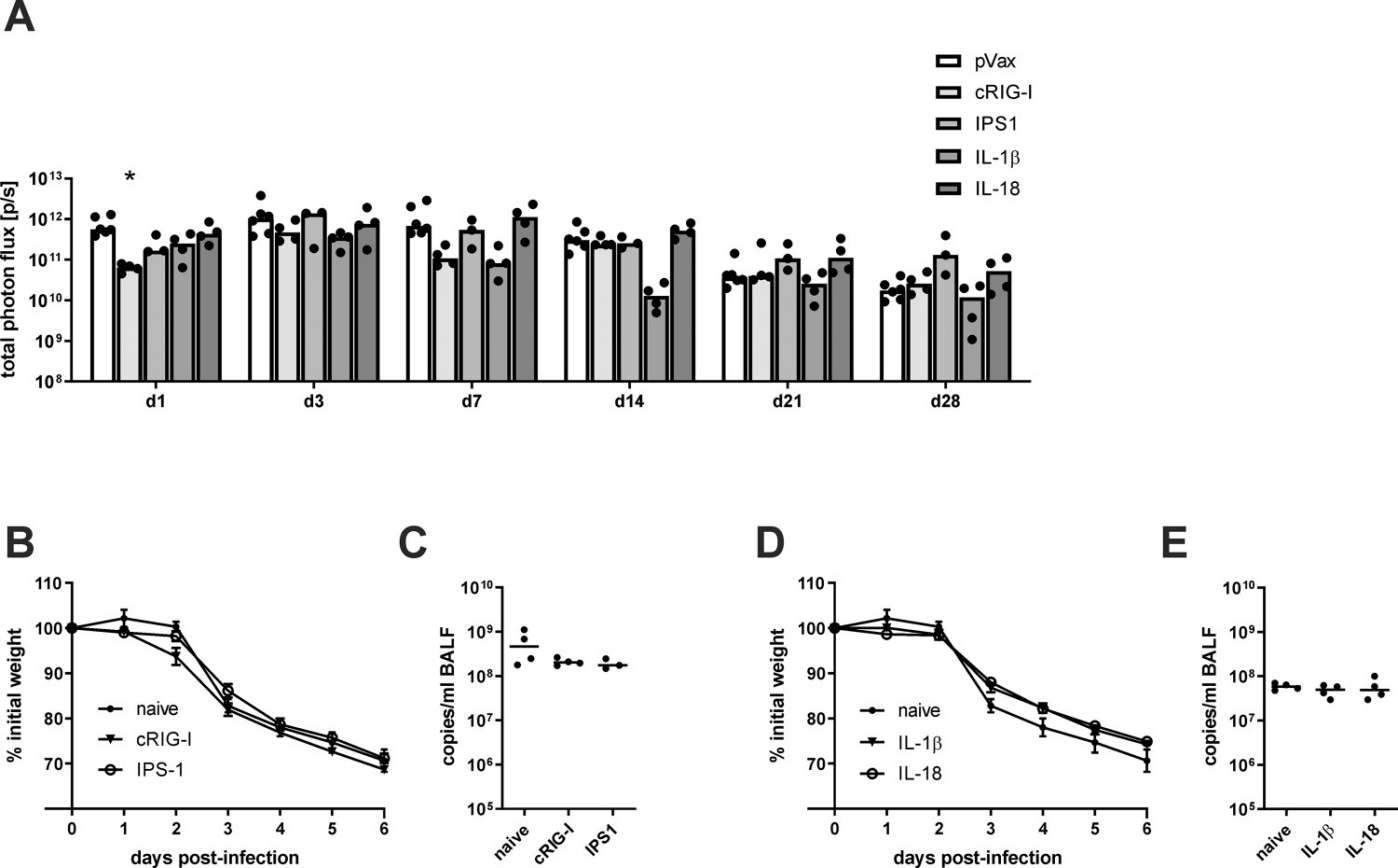
Antigen persistence and antigen-independent protection after adjuvant treatment. BALB/c mice were immunized with 20 μg luciferase-encoding plasmid and 10 μg adjuvant DNA (or pVax-empty as control) via intramuscular injection followed by electroporation. (A) At the indicated time points after immunization, 200 μg D-luciferin were injected into both legs and the luciferase signal was measured. The values for individual mice (mean of both legs) are shown and the bars represent the median for the group. Statistical significances were analysed by non-parametric two-way ANOVA followed by Dunnett's multiple comparisons test; *, p<0.05 vs. pVax-empty (n = 3–6 animals per group). (B-E) 35 days after the immunization, mice were infected with 250 PFU H1N1 PR8 and the weight loss was monitored daily (B+D). Six days post-infection, mice were euthanized, bronchoalveolar lavages were performed, and virus replication was analysed by qRT-PCR in those samples (C+E). Weight loss is depicted as mean ± SEM and the results of the qRT-PCR are displayed as individual data point for each animal and the group median (n = 3–4 animals per group).

Since the co-application of inflammatory adjuvants could potentially alter the antigen expression in terms of amount and duration, mice were immunized with 10 μg of a luciferase-encoding vector in combination with 10 μg of the adjuvant DNA (or pVax-empty). Between one and 28 days post-immunization, the luciferase activity was measured *in vivo* by local injection of D-Luciferin. In general, the luciferase activity peaked between day three and seven after immunization and then started to decline slowly but was still detectable after 28 days (Fig 2A). The cRIG-I group showed a significantly lower signal at the first time point but this was not recapitulated at other time points. Mice treated with IL-1β showed at seven and 14 days post treatment a lower luciferase activity but this trend did not reach statistical significance. Overall, there are no consistent differences in regard to the level and the duration of antigen expression between adjuvant-treated or control animals.

### Antigen-independent protective effects of the adjuvants

The previous experiment showed that expression of the DNA vaccine is able to persist for several weeks. To exclude that an ongoing production of the genetic adjuvants might affect the protection against a lethal IAV infection, mice that only received the respective adjuvant plasmid without viral antigens were infected with 250 PFU (25 LD_50_) H1N1 A/Puerto Rico/8/1934 (PR8). Importantly, neither the RIG-I- nor the NALP3-related adjuvants had an impact on the weight loss (Fig 2B and 2D) and viral replication (Fig 2C and 2E) compared to naive mice. Thus, at the time of the experimental IAV infection, the adjuvant treatment does not influence the course of an IAV infection in an antigen-unspecific manner.

### Immunogenicity of the adjuvants in systemic DNA immunizations

**Fig 3:**
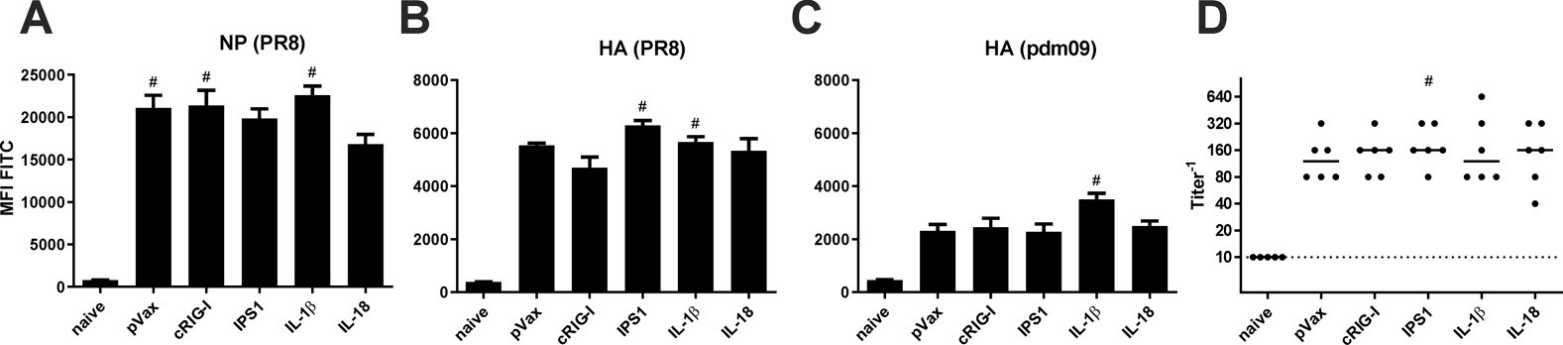
Humoral responses after DNA vaccination. BALB/c mice were immunized with 10 μg pVax-HA, 10 μg pVax-NP, and 10 μg adjuvant DNA (or pVax-empty as control) via intramuscular injection followed by electroporation. 28 days after the immunization, serum samples were generated from peripheral blood and the humoral response was analysed. (A-C) NP- and HA-expressing HEK 293T cells were incubated with serum dilutions (1:100 for NP and PR8 HA; 1:20 for pdm09 HA) and antigen-specific antibodies were measured with a FITC-conjugated detection antibody via flow cytometry. Values are depicted as mean + SEM for all groups and statistical significances were analysed by Kruskal-Wallis non-parametric one-way ANOVA followed by Dunn's post-test; #, p<0.05 vs. naive; *, p<0.05 vs. pVax-empty (n = 5–6 animals per group). (D) The neutralization capacity of serum samples was assessed by an *in vitro* neutralization assay against H1N1 PR8. Depicted are individual data points for each animal and the group median. The dotted line represents the detection limit (1:10 dilution). Statistical significances were analysed by Kruskal-Wallis non-parametric one-way ANOVA followed by Dunn's post-test; #, p<0.05 vs. naive; *, p<0.05 vs. pVax-empty (n = 5–6 animals per group).

To test the immunogenicity of the genetic adjuvants, we immunized mice with IAV antigen-encoding plasmids (10 μg pV-HA and 10 μg pV-NP) and the respective adjuvant plasmid (or pVax-empty as control; all 10 μg) by intramuscular injection followed by electroporation. 28 days later, blood samples were taken to analyse the vaccine-induced antibody responses. Using a FACS-based assay to detect antigen-binding antibodies, it was found that all immunization strategies induced humoral responses against NP and the homologous HA of the PR8 strain (Fig 3A and 3B). Statistically significant differences among the vaccine groups were not detected. However, the amount of antibodies able to bind a heterologous HA variant from pH1N1 A/Hamburg/4/2009 (pandemic isolate from 2009; pdm09) were slightly increased by the co-administration of IL-1β (Fig 3C). Moreover, all sera from immunized animals showed neutralizing activity against PR8, but the adjuvant treatments could not increase it further (Fig 3D). Importantly, although antibodies able to bind the HA of pdm09 were detected, none of the sera presented *in vitro* neutralization of this heterologous strain (S1 Fig).

**Fig 4:**
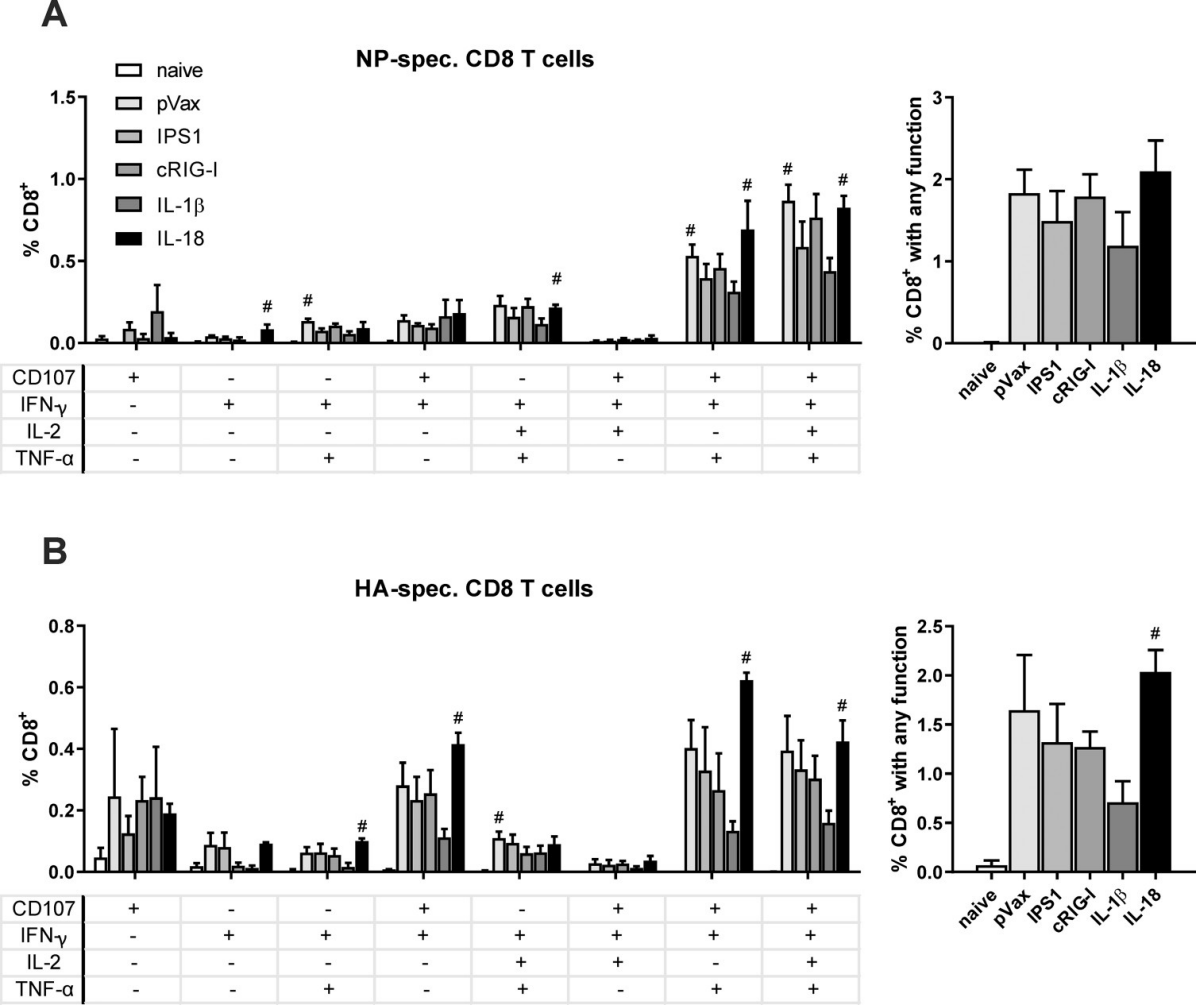
CD8^+^ T cell responses after vaccination. BALB/c mice were immunized with 10 μg pVax-HA, 10 μg pVax-NP, and 10 μg adjuvant DNA (or pVax-empty as control) via intramuscular injection followed by electroporation. Two weeks after the immunization, a subset of animals was euthanized to analyse the CD8^+^ T cell responses in the spleen. (A-B) Splenocytes were restimulated with the immunodominant MHC I restricted peptides from NP (NP_147-155_; A) or HA (HA_518-526_; B) and antigen-specific CD8^+^ T cells were identified by their secretion of IFN-γ, IL-2, and TNF-α as well as by the staining of CD107^+^ as degranulation marker. The left graphs depict mono- and polyfunctional populations, while the right graphs show the sum of responsive antigen-specific T cells positive for at least one of the functional marker. Mean values with standard error of the mean (SEM) represent data from four mice per group. #, p < 0.05 vs. naive; *, p < 0.05 vs. pVax-empty (Kruskal-Wallis non-parametric one-way ANOVA followed by Dunn's post-test).

Next, we assessed the vaccine-induced CD8^+^ T cell responses against the viral antigens. To this end, we isolated splenocytes two weeks after the immunization and restimulated them *ex vivo* with immunodominant MHC class I restricted peptides (NP_147-155_ or HA_518-526_). The NP-specific response consisted predominantly of polyfunctional T cells showing production of IFN-γ and TNF-α as well as degranulation (indicated by CD107a staining) upon restimulation, while about half of those cells produced IL-2 in addition (Fig 4A). The HA-specific CD8^+^ T cell response was also dominated by polyfunctional T cells and in addition significant proportions of CD107^+^IFN-γ^+^ as well as CD107^+^ cells were observed (Fig 4B). Regarding the adjuvant treatments, no significant differences among the immunized groups could be observed. However, there was a trend to a decreased responsiveness of CD8^+^ T-cells for both antigen-specificities when IL-1β was co-delivered as adjuvant. In addition, the CD4^+^ T cell responses against a MHC class II restricted peptide derived from HA were analysed but showed only minor responses without significant improvements by the adjuvant treatments (S2 Fig). In conclusion, none of the adjuvants had a beneficial impact on the immunogenicity.

### Protective efficacy against homologous and heterologous IAV strains

**Fig 5:**
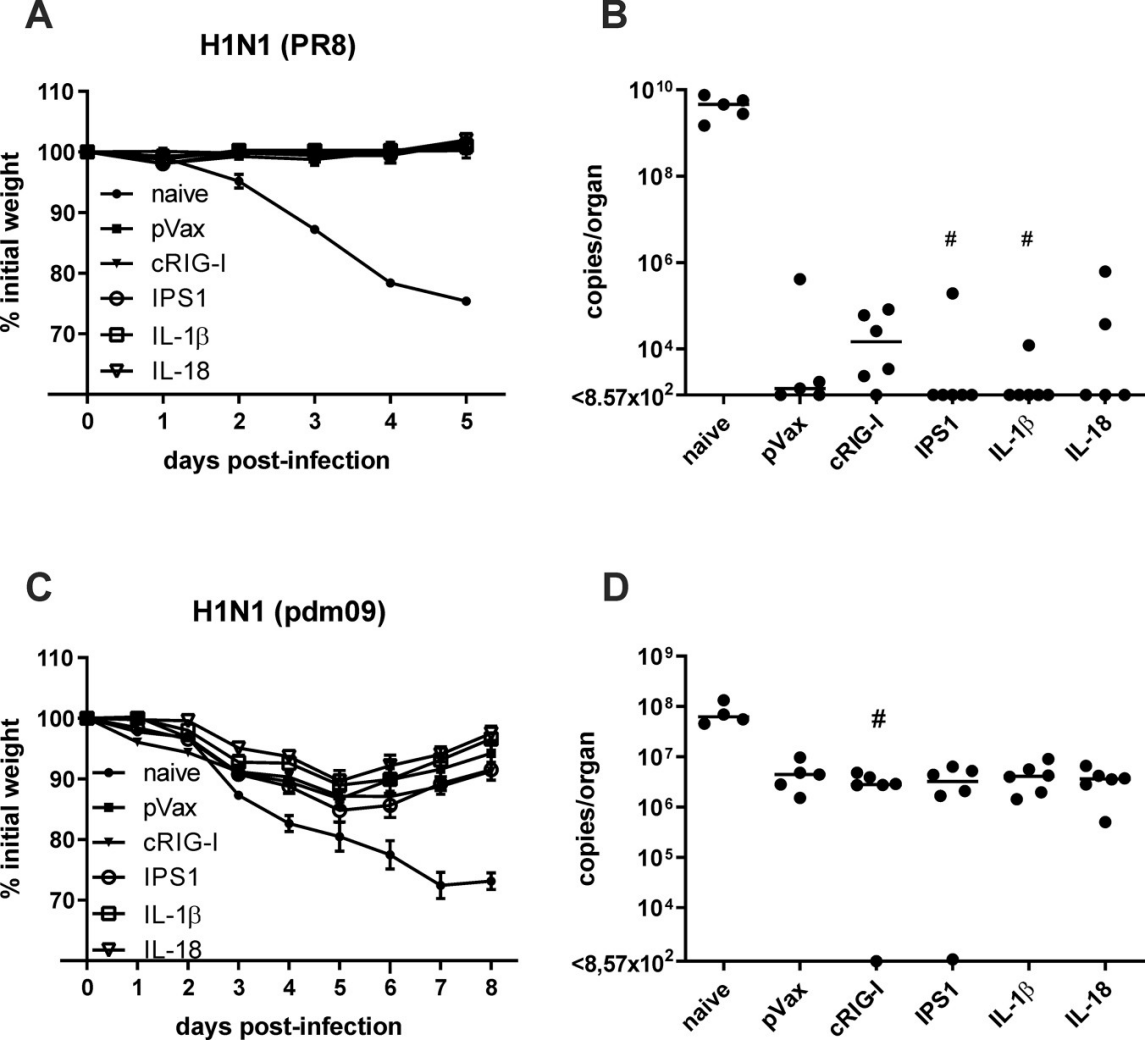
Protective efficacy against influenza infections. BALB/c mice were immunized with 10 μg pVax-HA, 10 μg pVax-NP, and 10 μg adjuvant DNA (or pVax-empty as control) via intramuscular injection followed by electroporation. 35 days after the immunization, mice were infected with 2500 PFU H1N1 PR8 (A) or 5000 PFU pdm09 (B) and the weight loss was monitored daily (A+C). Five (B) and eight days (D) post-infection, mice were euthanized, lungs were harvested, and virus replication was analysed by qRT-PCR in those samples (B+D). Weight loss is depicted as mean ± SEM and the results of the qRT-PCR are displayed as individual data point for each animal and the group median (n = 4–6 animals per group). #, p < 0.05 vs. naive (Kruskal-Wallis non-parametric one-way ANOVA followed by Dunn's post-test). No statistically significant differences between pVax-empty and the adjuvanted groups were found.

In order to evaluate the protective efficacy of the vaccination strategies, mice were infected with a lethal dose of H1N1 PR8 35 days after immunization (2500 PFU; 250 LD_50_). Probably due to the high neutralizing activity against PR8, the immunized animals did not experience any weight loss upon infection (Fig 5A) and low virus replication in the lung (Fig 5B). Six-teen out of 28 immunized mice showed no detectable virus replication at all, while naive animals lost more than 20% weight and showed uncontrolled virus replication in the lung. Thus, the DNA vaccine provided rapid and efficient control against the homologous infection but from these data, we could not interpret any differences due to the adjuvant inclusion.

Next, the protective capacity against a heterologous IAV strain was investigated. 35 days post immunization, mice were challenged with a lethal dose of pdm09 (5000 PFU; 10 LD_50_). Naive animals lost almost 30% weight and reached the final end point criteria within seven days (Fig 5C). In contrast, morbidity was observable in all immunized groups as well but peaked at 5 days post-infection with around 10% weight loss before the mice started to recover. However, no positive effects of the adjuvant treatments were found. Similarly, virus replication was decreased by the factor of ten in the immunized mice compared to the naive group, but most of the animals showed highly similar viral loads in the lungs (Fig 5D). Somewhat unexpected, we were not able to detect such viral loads in the lungs (Fig 5D) and BALF (S3 Fig) of one cRIG-I- and one IPS1-treated animal, yet both animals showed significant weight loss during infection. In conclusion, the intramuscular DNA vaccination against HA and NP provided efficient protection against infections with the homologous and a heterologous IAV strain but did not reveal any positive effect through the inclusion of the genetic adjuvants.

### Immunogenicity of combined immunizations with two adjuvants

**Fig 6:**
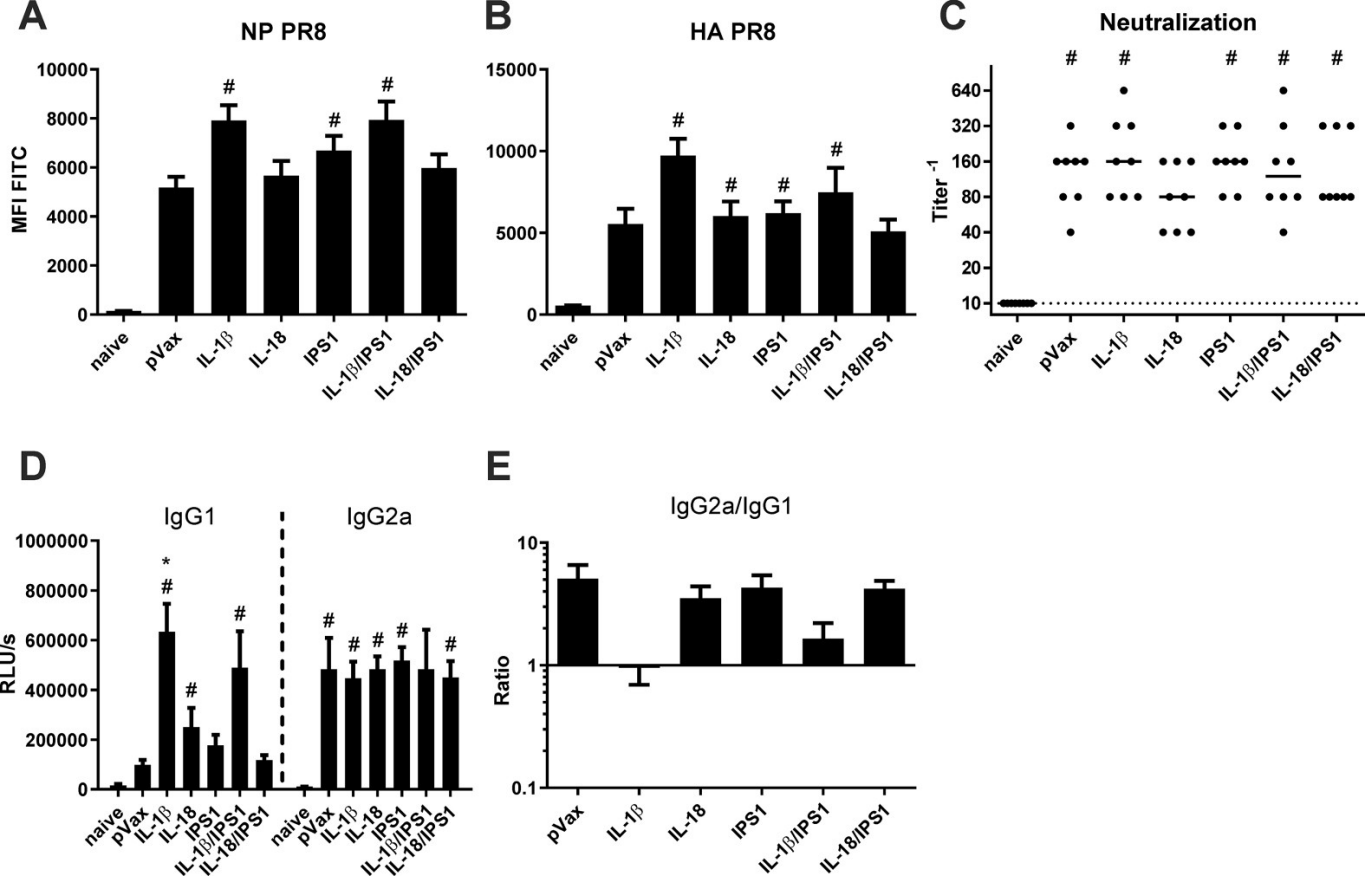
Humoral responses after DNA vaccination with combined adjuvants. BALB/c mice were immunized with 5 μg pVax-HA, 5 μg pVax-NP, and 10 μg of each adjuvant DNA via intramuscular injection followed by electroporation. 28 days after the immunization, serum samples were generated from peripheral blood and the humoral response was analysed. (A-B) NP- and HA-expressing HEK 293T cells were incubated with 1:100 serum dilutions and antigen-specific antibodies were measured with a FITC-conjugated detection antibody via flow cytometry. Values are depicted as mean + SEM for all groups and statistical significances were analysed by Kruskal-Wallis non-parametric one-way ANOVA followed by Dunn's post-test; #, p<0.05 vs. naive; *, p<0.05 vs. pVax-empty (n = 7–8 animals per group). (C) The neutralization capacity of serum samples was assessed by an *in vitro* neutralization assay against H1N1 PR8. Depicted are individual data points for each animal and the group median. The dotted line represents the detection limit (1:10 dilution). Statistical significances were analysed by Kruskal-Wallis non-parametric one-way ANOVA followed by Dunn's post-test; #, p<0.05 vs. naive; *, p<0.05 vs. pVax-empty (n = 7–8 animals per group). (D-E) IAV-specific IgG1 and IgG2a were analysed by an antigen-specific antibody ELISA with coated the PR8 strain. Serum samples were measured in a 1:100 dilution and mean values with SEM of the relative light units (RLU) is shown (D). In (E) the IgG2a/IgG1 ratios are depicted. Statistical significances were analysed by Kruskal-Wallis non-parametric one-way ANOVA followed by Dunn's post-test; *, p<0.05 vs. pVax-empty (n = 7–8 animals per group).

Since the previous experiments did not expose an increased immunogenicity or enhanced protective efficacy by the separate inclusion of RIG-I- or NALP3-related adjuvants, we wanted to combine signalling molecules of both pathways to analyse whether any synergistic effects occur. Therefore, pV-IPS1 was co-administered with pV-IL-1β and pV-IL-18, respectively, and this treatment was compared to single adjuvant treatments and to a pVax-empty control group. This time, the animals were immunized with a decreased dose of antigen-encoding vectors (pV-HA and pV-NP, 5 μg each) and 10 μg per adjuvant plasmid. Single adjuvant groups got 10 μg pVax-empty instead of the second adjuvant. Antibody responses were analysed and displayed again an efficient induction of antibodies against the vaccine antigens NP (Fig 6A) and HA (Fig 6B) in all immunized animals. Interestingly, with the decreased antigen dose, antibody responses were slightly increased in mice that received pV-IL-1β, both as single adjuvant and in combination with pV-IPS1. The *in vitro* neutralization assay did not reflect these findings (Fig 6C). The increase of HA-specific antibodies was probably too small to increase neutralization to the next titration step (at least 2-fold increase needed). An IgG subtype specific ELISA indicated that the increase of antigen-specific antibodies in the IL-1β-treated groups was most probably due to a specific increase of IgG1 and thereby yielded a decreased IgG2a/IgG1 ratio (Fig 6D and 6E). However, synergistic effects by the combination of adjuvants were not observed with any of these methods.

**Fig 7:**
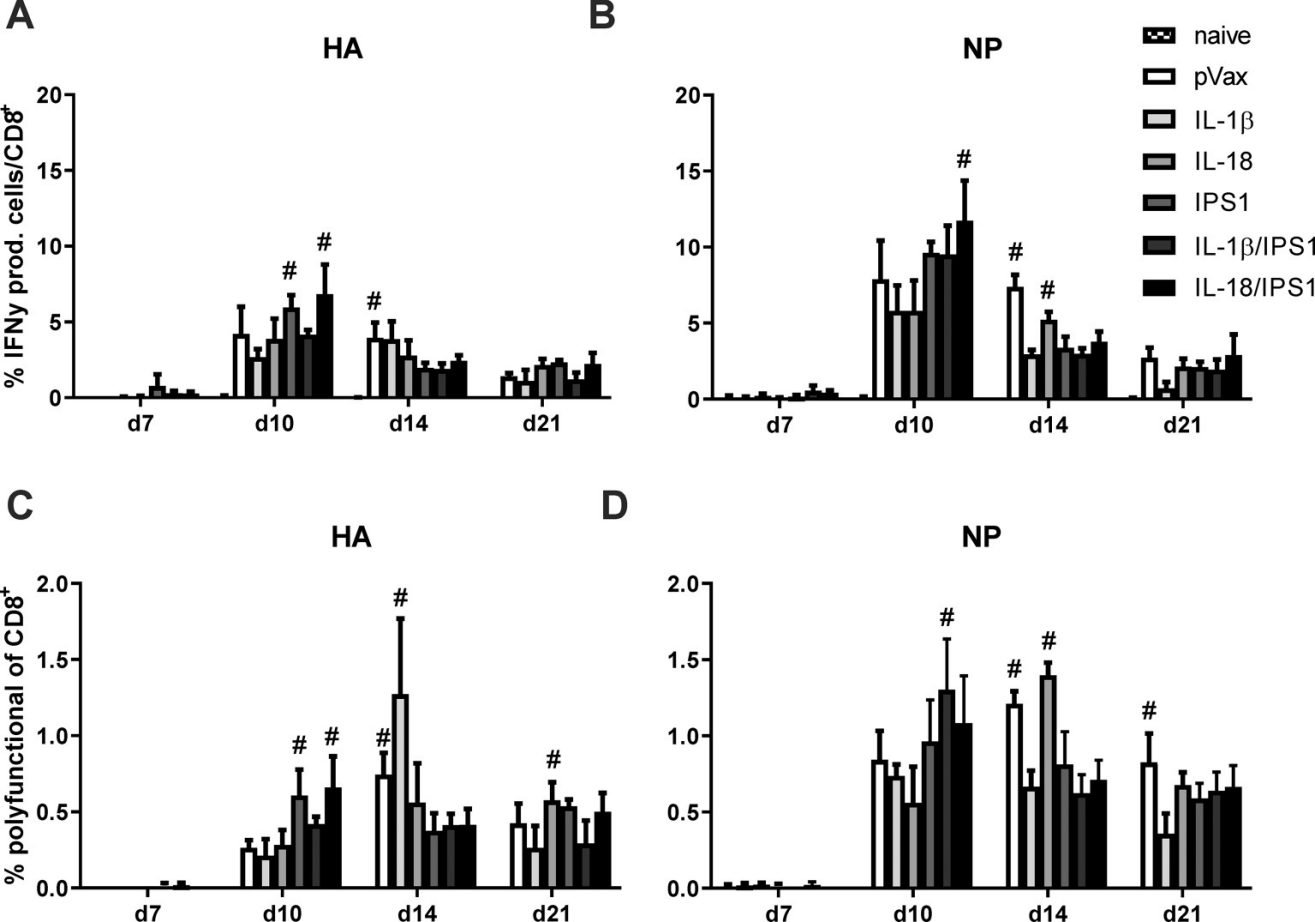
CD8^+^ T cell kinetic after DNA vaccination with combined adjuvants. BALB/c mice were immunized with 5 μg pVax-HA, 5 μg pVax-NP, and 10 μg of each adjuvant DNA via intramuscular injection followed by electroporation. At the indicated time points after immunization, peripheral blood was drawn to analyse the CD8^+^ T cell response. (A-B) Lymphocytes were restimulated with the immunodominant MHC I restricted peptides from NP (NP_147-155_; B+D) or HA (HA_518-526_; A+C) and antigen-specific CD8^+^ T cells were identified by their secretion of IFN-γ, IL-2, and TNF-α as well as by the staining of CD107^+^ as degranulation marker. Depicted is the percentage of all CD8^+^ T cells that express IFN-γ (independent of other cytokines; A+B) and the percentage of CD8^+^ T cells that show a polyfunctional profile (CD107^+^IFN-γ^+^TNF-α^+^IL-2^+^; C+D). Mean values with standard error of the mean (SEM) represent data from four mice per group. #, p < 0.05 vs. naive; *, p < 0.05 vs. pVax-empty (Kruskal-Wallis non-parametric one-way ANOVA followed by Dunn's post-test).

Next, the immunogenicity was assessed in terms of CD8^+^ T cell responses. We wanted to assure not to miss transient adjuvant effects and therefore analysed the responses longitudinally between day 7 and day 21 in peripheral blood. For reasons of simplicity, Fig 7 depicts all antigen-specific CD8^+^ T cells that respond with IFN-γ (Fig 7A and 7B) or that respond with a polyfunctional profile (CD107^+^IFN-γ^+^IL-2^+^TNFα^+^; Fig 7C and 7D). While the total pool of IFN-γ-producing cells peaks around day 10 and then steadily declined in size, the polyfunctional population seemed much more stable over time, presumable reflecting the phenotypic differences between short-lived effector cells and memory T cells. Regarding the different adjuvant strategies, neither HA-specific (Fig 7A and 7C) nor NP-specific responses (Fig 7B and 7D) showed any statistically significant differences among the immunized groups. Thus, none of the genetic adjuvants improved the CD8^+^ T cell responses significantly.

### Heterologous protection of combined immunizations with two adjuvants

**Fig 8:**
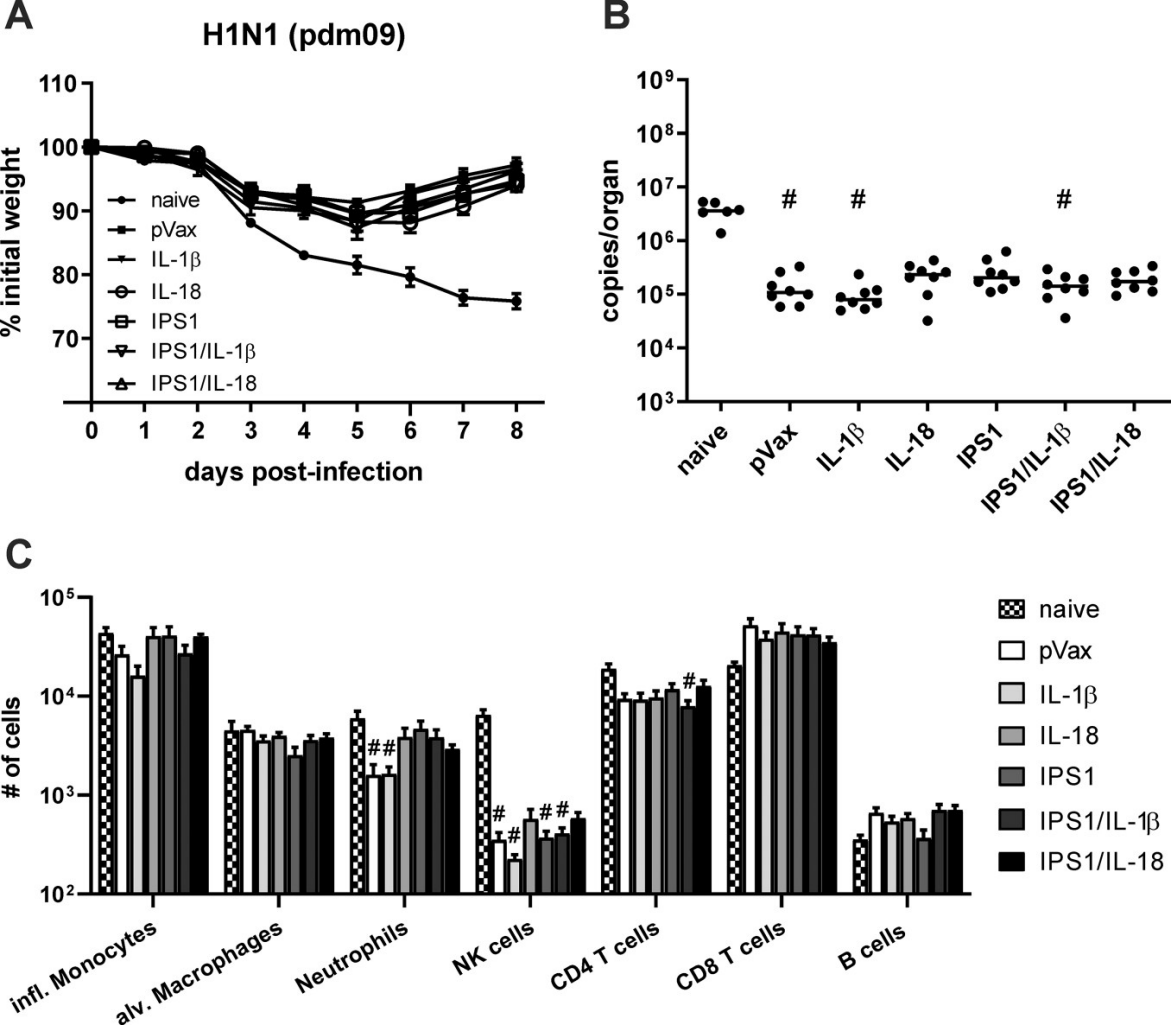
Protective efficacy after DNA vaccination with combined adjuvants. BALB/c mice were immunized with 5 μg pVax-HA, 5 μg pVax-NP, and 10 μg of each adjuvant DNA via intramuscular injection followed by electroporation. 35 days after the immunization, mice were infected with 5000 PFU pH1N1 pdm09 and the weight loss was monitored daily (A). Eight days post-infection, mice were euthanized, bronchoalveolar lavages were performed, and lungs were harvested. Virus replication was analysed by qRT-PCR in lung homogenates (B) and infiltrated immune cells in BAL fluids were assessed by flow cytometry (C). Weight loss is depicted as mean ± SEM, the results of the qRT-PCR are displayed as individual data point for each animal and the group median, and the cellular infiltration is shown as mean with SEM (n = 6–8 animals per group). #, p < 0.05 vs. naive; *, p < 0.05 vs. pVax-empty (Kruskal-Wallis non-parametric one-way ANOVA followed by Dunn's post-test).

Next, the mice were infected with a lethal dose of the heterologous IAV strain pdm09 (5000 PFU; 10 LD_50_) to analyse the heterologous immunity. Again, the immunized mice showed an initial weight loss but started to recover around day five or six post-infection (Fig 8A). The weight curves among the immunized groups showed no significant differences. Similarly, viral RNA copies were about 50- to 100-fold lower in immunized compared to naive mice, but there was no influence of single or combined adjuvant co-administrations on the virus replication (Fig 8B). In addition, we assessed the presence of immune cells in BALF samples at day eight post-infection to get insights into the presence of adaptive and inflammatory cell types. Concerning the adaptive immune cells, there were no significant differences in the infiltration of T and B cells among the immunized groups but in general naive animals tended to show a more pronounced infiltration of CD4^+^ T cells and less CD8^+^ T cells as well as B cells (Fig 8C). Similarly, high numbers of NK cells infiltrated the lungs of naive mice while found in substantial lower numbers in immunized animals but without quantitative differences among different adjuvant treatments. Inflammatory monocytes, alveolar macrophages, and neutrophils were present in comparable numbers among naive and immunized groups, but the latter ones tended to be less frequent in the pVax and IL-1β treatment groups. Altogether, the experimental infection did not provide any evidence for an increased heterologous protection by the single or combined administration of genetic adjuvants.

### *In vivo* assessment of vaccine-induced inflammation

**Fig 9:**
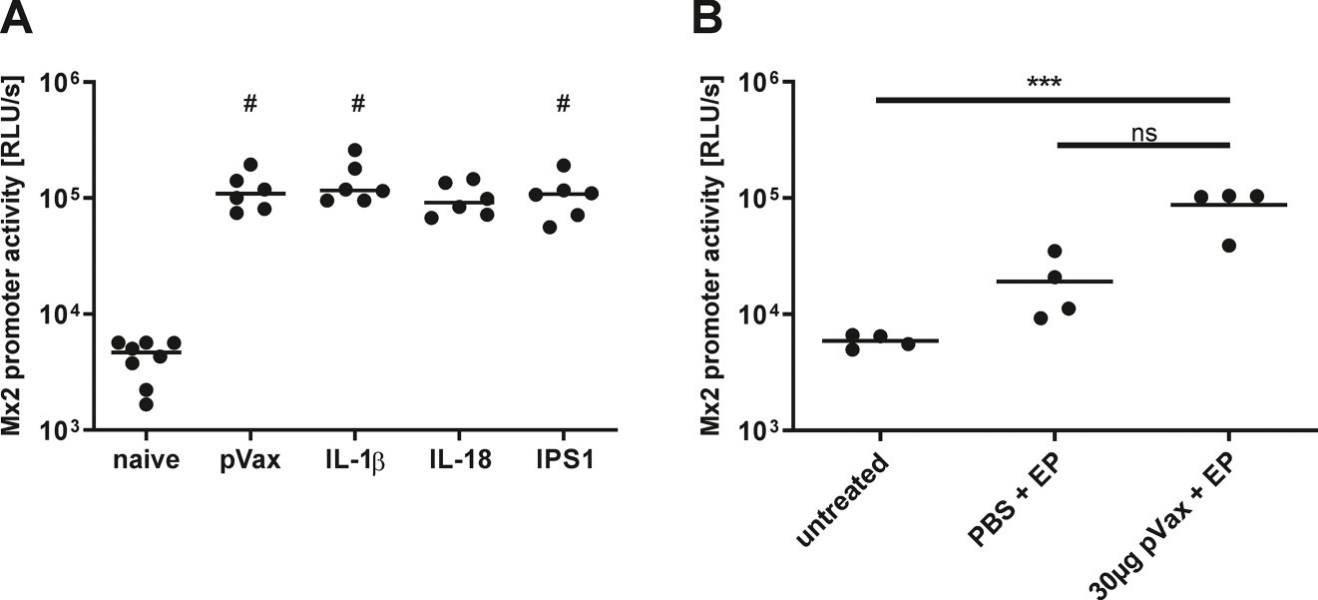
*In vivo* inflammation after vaccinations. Mx2-Luc reporter mice were immunized with 10 μg of the respective plasmid via intramuscular injection followed by electroporation (A) or were treated as depicted in the graph (B). 72 hours later, the electroporated muscles were excised and homogenized. The luciferase signal was analysed in the muscle homogenate as a surrogate for Mx2 promoter activity. Depicted are individual data points from each animal (two legs per mouse measured) and the group median (2–4 animals per group). #, p < 0.05 vs. naive; ***, p < 0.001 (Kruskal-Wallis non-parametric one-way ANOVA followed by Dunn's post-test). RLU/s, relative light units per second.

We were not able to demonstrate any consistent adjuvant effect in our DNA vaccination setting beside slight effects of pV-IL-1β on antigen persistence and IgG1 induction. Although the *in vitro* bioactivity of the adjuvants was verified in the beginning, we wondered whether this bioactivity is also present *in vivo*. Therefore, transgenic Mx2-Luc mice were exploited, which express the firefly luciferase under the control of the type I and type III IFN-inducible Mx2 promoter [56], to investigate the induction of type I and III IFN as a surrogate for *in vivo* inflammation. Mx2-Luc mice were immunized with 10 μg pVax-empty or the respective amount adjuvant plasmid and 72 hours later, the luciferase signal was determined in whole muscle lysates. Compared to naive mice, an increased luciferase activity was detected in all mice that received a DNA immunization (Fig 9A). However, the signals were comparable among all immunized animals. This led us to the speculation that the administered DNA (independent of the insert) together with the electroporation already present a quite inflammatory stimulus and this could perhaps provide redundancy to the adjuvant-specific effects. We further underlined this hypothesis by demonstrating that the electroporation without DNA and the electroporation with pVax-empty each resulted in an incremental increase of the Mx2 promoter activity (Fig 9B).

## Discussion

Recent vaccination strategies against seasonal influenza epidemics possess several major drawbacks like their antiquated production process, the need for a yearly vaccine update, and their poor immunogenicity in the elderly. Most importantly, recent vaccines induce only strain-specific protection against IAV strains expected to circulate in the upcoming flu season. In case of a vaccine mismatch to the predominantly circulating strain in an epidemic or pandemic scenario, those vaccines do not provide any protection [4]. Thus, novel vaccination strategies that establish a broad and long-lasting protection are urgently needed.

In the present study, we employed systemic DNA immunizations against IAV with genetic adjuvants derived from innate immune pathways. In particular, we tested IPS1 and constitutively active RIG-I from the RIG-I pathway and IL-1β as well as IL-18 from the NALP3 inflammasome pathway.

All adjuvant-encoding vaccine plasmids were tested *in vitro* and showed distinct bioactivity profiles. Unfortunately, the RIG-I related adjuvants did not improve the immunogenicity or protective efficacy of the DNA vaccination compared to a non-coding adjuvant plasmid. As described in previous studies [17,19,21,22,54,55], the DNA vaccine protected mice efficiently against mortality during homologous and heterologous IAV infections *per se*, but no improvements were observed due to the adjuvant treatments. This was unexpected because other studies demonstrated activation of the RIG-I axis as a promising target to increase immunogenicity of DNA vaccines in BALB/c mice. In particular, Luo and colleagues co-immunized with IPS1-encoding DNA (without electroporation) and observed increased cellular but not humoral responses in mice [57]. Moreover, Luke et al. used DNA plasmids encoding for IAV antigens as well as encoding RIG-I agonists and reported increased humoral responses after naked DNA injection but not after additional electroporation [35]. These studies already indicate that activation of the RIG-I axis can potentially increase immunogenicity in naked DNA injections, but additional electroporation might provide a redundant inflammatory stimulus to the specific RIG-I axis activation. Indeed, our results demonstrate that electroporation without DNA induces Mx2 promoter activation *in vivo*, while electroporation with non-coding DNA further increases this inflammation, probably by activation of innate DNA sensors like AIM2 [58] or cGAS-STING [59]. However, the addition of genetic adjuvants did not enhance the IFN-induced luciferase expression in these mice, although the signalling molecules were highly potent activators of type I IFN *in vitro*. Thus, we cautiously hypothesize that innate DNA recognition plus the electroporation-induced tissue damage might already provide a sufficient inflammation and therefore adjuvant-related inflammation could be redundant. This is in line with other studies that also reported incremental contributions of injection traumata, DNA sensing, and electroporation to tissue inflammation in the treated muscle characterized by rhabdomyolysis as well as infiltration of mast cells, eosinophils, macrophages, dendritic cells, B cells, and T cells [60,61]. Interestingly, Liu and colleagues also tested genetic adjuvants, specifically DNA-encoded Flt3L and MIP-1α, and found that either electroporation or inclusion of genetic adjuvants can both increase immunogenicity of the DNA vaccine separately, but if electroporation was applied after the injection of the adjuvants, an additive effect was barely detectable [61]. However, this might not rule out that adjuvants addressing other pathways might possess better immunostimulatory potentials in DNA electroporation [62,63] or that the adjuvants might have greater impact in immunizations with immunosuppressive antigens [64].

Similar to the RIG-I related adjuvants, neither IL-1β nor IL-18 increased the protective efficacy of our DNA vaccine. However, IL-1β increased antibody levels in low dose immunizations and in particular murine IgG1. As demonstrated by others, this might be the consequence of an increased CD4 T_H_2 expansion [45] and the absence of IL-1 receptor 1 expression on T_H_1 cells [65]. HA-specific antibody responses mostly play a role in infections with the homologous IAV strain by neutralizing the virus before cell entry. However, the increased IgG1 amount did not translate in elevated *in vitro* virus neutralization. In regard to secondary effector functions, murine IgG1 has a low capacity to engage activating Fc receptors and therefore should probably not enhance functions like ADCC *in vivo* [66]. In regard to the protection against heterologous IAV strains, T cell responses specific for conserved viral proteins can suppress viral replication and thereby reduce morbidity and mortality upon heterologous infection [11–14]. NP is such a conserved viral antigen of IAV and therefore NP-specific T cell responses were evaluated in this study. However, there was no substantial influence of plasmid-encoded IL-1β observed and in line with that, the heterologous protection was not affected. In a former study, we used IL-1β as genetic adjuvant in intranasal immunizations with adenoviral vectors against IAV. There, IL-1β revealed strong adjuvant effects and induced an increased protection against several IAV strains [21]. In particular, it strongly increased the induction of tissue-resident memory CD8^+^ T cells in the lung. Similar to the present study, intramuscular application of the viral vector encoding IL-1β did not enhance immunogenicity. Therefore, we claim that IL-1β is a strict mucosal adjuvant with only low adjuvant potential in intramuscular administrations.

In theory, IL-18 should activate a highly similar pathway to IL-1β signalling [67], but in our studies we could not observe any *in vivo* effects despite *in vitro* bioactivity. One explanation could be that the IL-18 receptor beta subunit is only expressed in some cell types and its absence in muscle tissue could thereby prevent *in vivo* activity [67]. Moreover, it is reported that the bioactivity of IL-18 requires synergy with IL-12 [68]. In addition, as argued above, the DNA injection and electroporation might activate innate pathways like AIM2, which initiate IL-1β/IL-18 expression as well, and thereby the additional expression of those cytokines might not provide an additional benefit. In contrast to our results, others observed adjuvant effects of IL-18 in DNA immunizations in different viral, bacterial, or malignant disease models [51,69–73]. In particular, almost all studies reported a moderate enhancement of T_H_1 responses. However, since electroporation was used in none of these studies as immunization technique, those results do not contradict our interpretation.

In conclusion, plasmid-encoded cRIG-I, IPS1, IL-1β, and IL-18 did not improve the immunogenicity or protective efficacy of a DNA vaccine against IAV in the context of electroporation. We suspect that the innate DNA sensing pathways in combination with the electroporation provide an almost optimal inflammation, at least in mice, which makes the adjuvants in our setting redundant. However, our study provides once again evidence that genetic vaccines can induce protective immunity against homologous and heterologous IAV strains.

## Supporting information

S1 Fig Neutralization of pdm09 strain. BALB/c mice were immunized with 10 μg pVax-HA, 10 μg pVax-NP, and 10 μg adjuvant DNA (or pVax-empty as control) via intramuscular injection followed by electroporation. 28 days after the immunization, serum samples were generated from peripheral blood and the humoral response was analysed. The neutralization capacity of serum samples was assessed by an *in vitro* neutralization assay against pH1N1 pdm09 A/Hamburg/4/2009. Depicted are individual data points for each animal and the group median. The dotted line represents the detection limit (1:10 dilution). n = 5–6 animals per group.
(DOCX)

S2 Fig CD4^+^ T cell responses after vaccination. BALB/c mice were immunized with 10 μg pVax-HA, 10 μg pVax-NP, and 10 μg adjuvant DNA (or pVax-empty as control) via intramuscular injection followed by electroporation. Two weeks after the immunization, a subset of animals was euthanized to analyse the CD4^+^ T cell responses in the spleen. Splenocytes were restimulated with the immunodominant MHC II restricted peptide from HA (HA_110-120_) and antigen-specific CD4^+^ T cells were identified by their secretion of IFN-γ, IL-2, and TNF-α. Mean values with standard error of the mean (SEM) represent data from four mice per group. #, p < 0.05 vs. naive; *, p < 0.05 vs. pVax-empty (Kruskal-Wallis non-parametric one-way ANOVA followed by Dunn's post-test).
(DOCX)

S3 Fig qPCR analysis of viral RNA in BALF. BALB/c mice were immunized with 5 μg pVax-HA, 5 μg pVax-NP, and 10 μg total adjuvant DNA via intramuscular injection followed by electroporation. 35 days after the immunization, mice were infected with 5000 PFU pH1N1 A/Hamburg/4/2009. Eight days post-infection, mice were euthanized, bronchoalveolar lavages were performed, and virus replication was analysed by qRT-PCR in BALF. The results of the qRT-PCR are displayed as individual data point for each animal and the group median (n = 4–6 animals per group). #, p < 0.05 vs. naive; *, p < 0.05 vs. pVax-empty (Kruskal-Wallis non-parametric one-way ANOVA followed by Dunn's post-test).
(DOCX)

S1 Raw Data (XLSX)
