# Supplementary figures and images for: Innate signalling molecules as genetic adjuvants do not alter the efficacy of a DNA-based influenza A vaccine
Source: PLoS One. 2020 Apr 3;15(4):e0231138. doi: 10.1371/journal.pone.0231138 (PMC7122823; doi:10.1371/journal.pone.0231138)

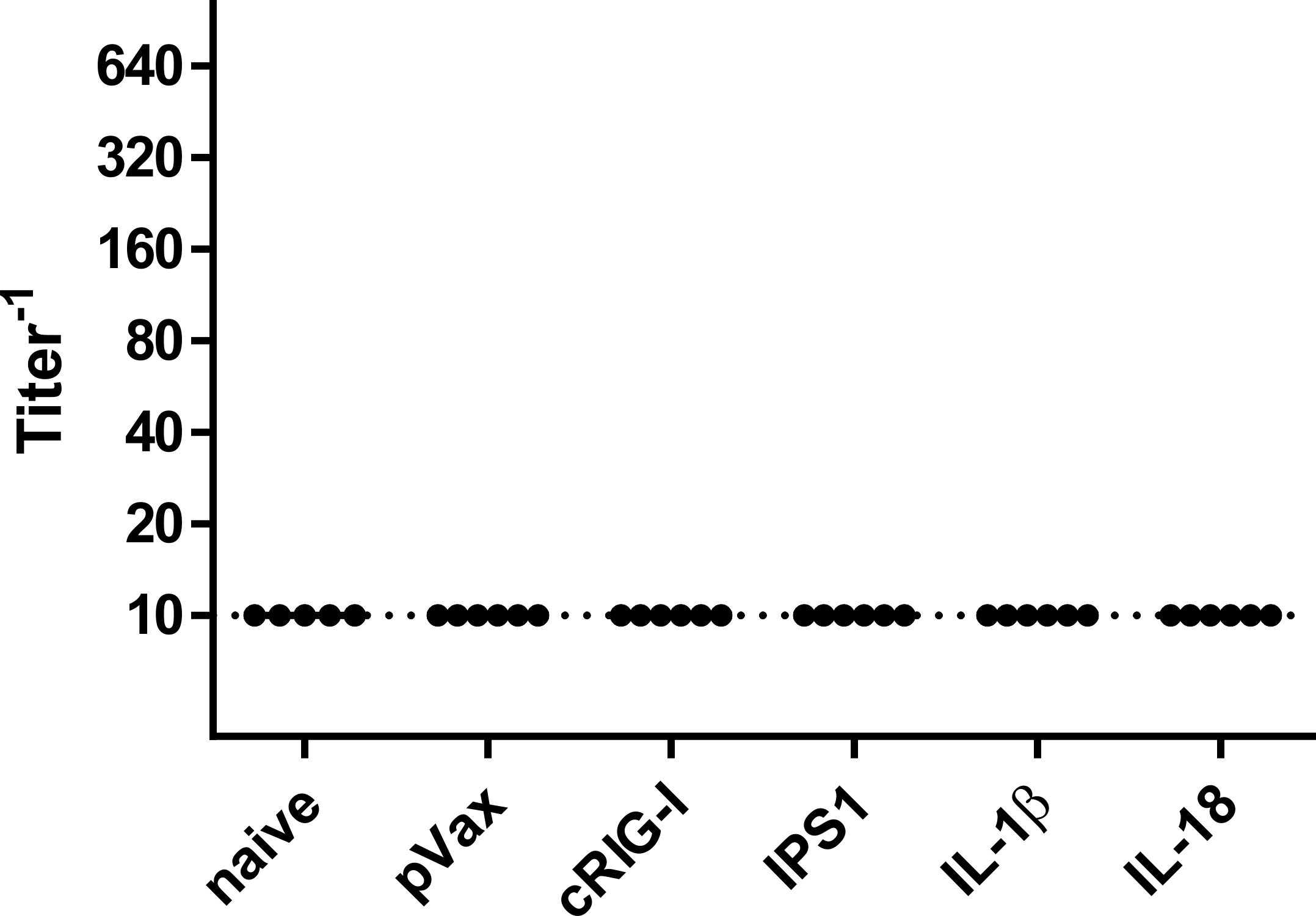


**Fig. S1: Neutralization of pdm09 strain.**

Supplement: S1 Fig — BALB/c mice were immunized with 10 μg pVax-HA, 10 μg pVax-NP, and 10 μg adjuvant DNA (or pVax-empty as control) via intramuscular injection followed by electroporation. 28 days after the immunization, serum samples were generated from peripheral blood and the humoral response was analysed. The neutralization capacity of serum samples was assessed by an in vitro neutralization assay against pH1N1 pdm09 A/Hamburg/4/2009. Depicted are individual data points for each animal and the group median. The dotted line represents the detection limit (1:10 dilution). n = 5–6 animals per group. (DOCX) [file pone.0231138.s001.docx]

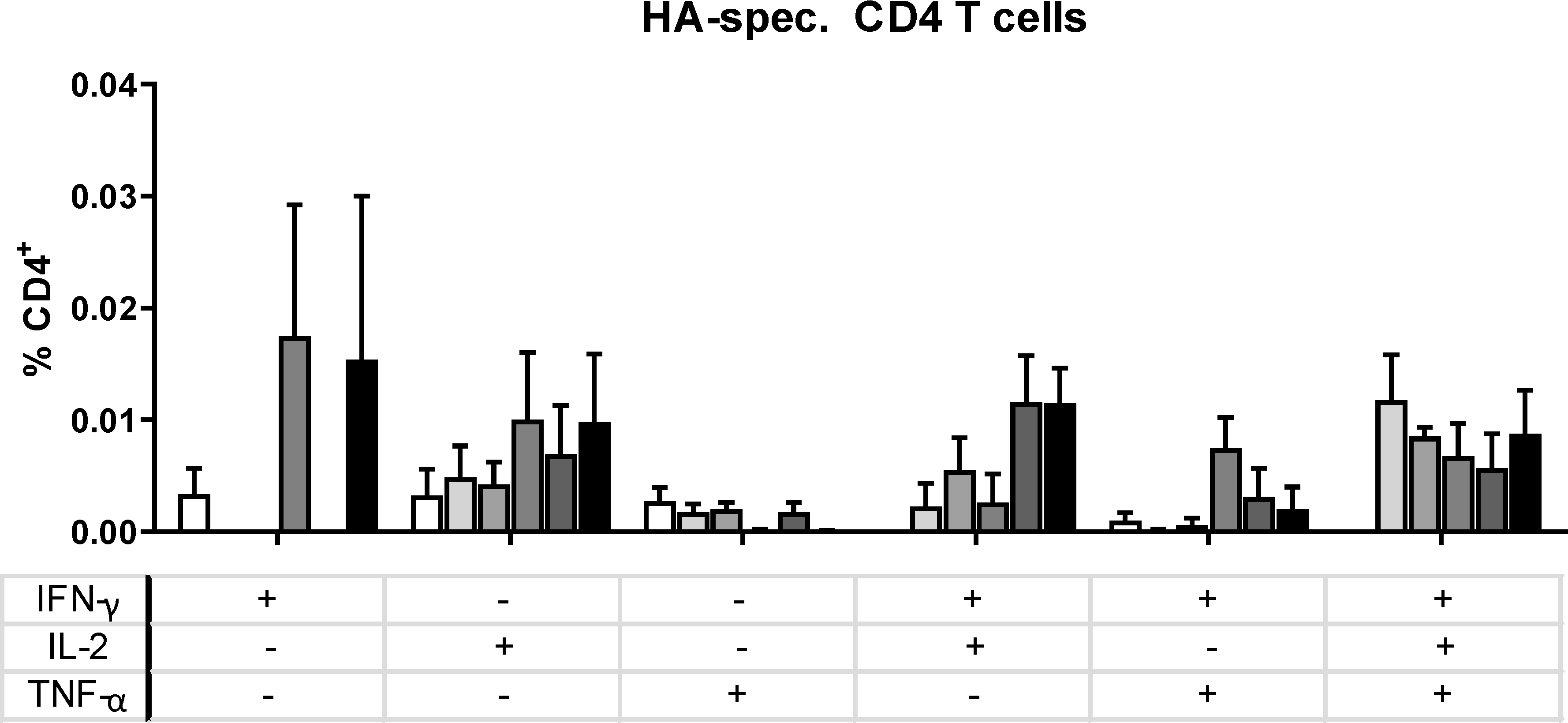


**Fig. S2: CD4+ T cell responses against HA110-120.**

Supplement: S2 Fig — BALB/c mice were immunized with 10 μg pVax-HA, 10 μg pVax-NP, and 10 μg adjuvant DNA (or pVax-empty as control) via intramuscular injection followed by electroporation. Two weeks after the immunization, a subset of animals was euthanized to analyse the CD4+ T cell responses in the spleen. Splenocytes were restimulated with the immunodominant MHC II restricted peptide from HA (HA110-120) and antigen-specific CD4+ T cells were identified by their secretion of IFN-γ, IL-2, and TNF-α. Mean values with standard error of the mean (SEM) represent data from four mice per group. #, p < 0.05 vs. naive; *, p < 0.05 vs. pVax-empty (Kruskal-Wallis non-parametric one-way ANOVA followed by Dunn's post-test). (DOCX) [file pone.0231138.s002.docx]

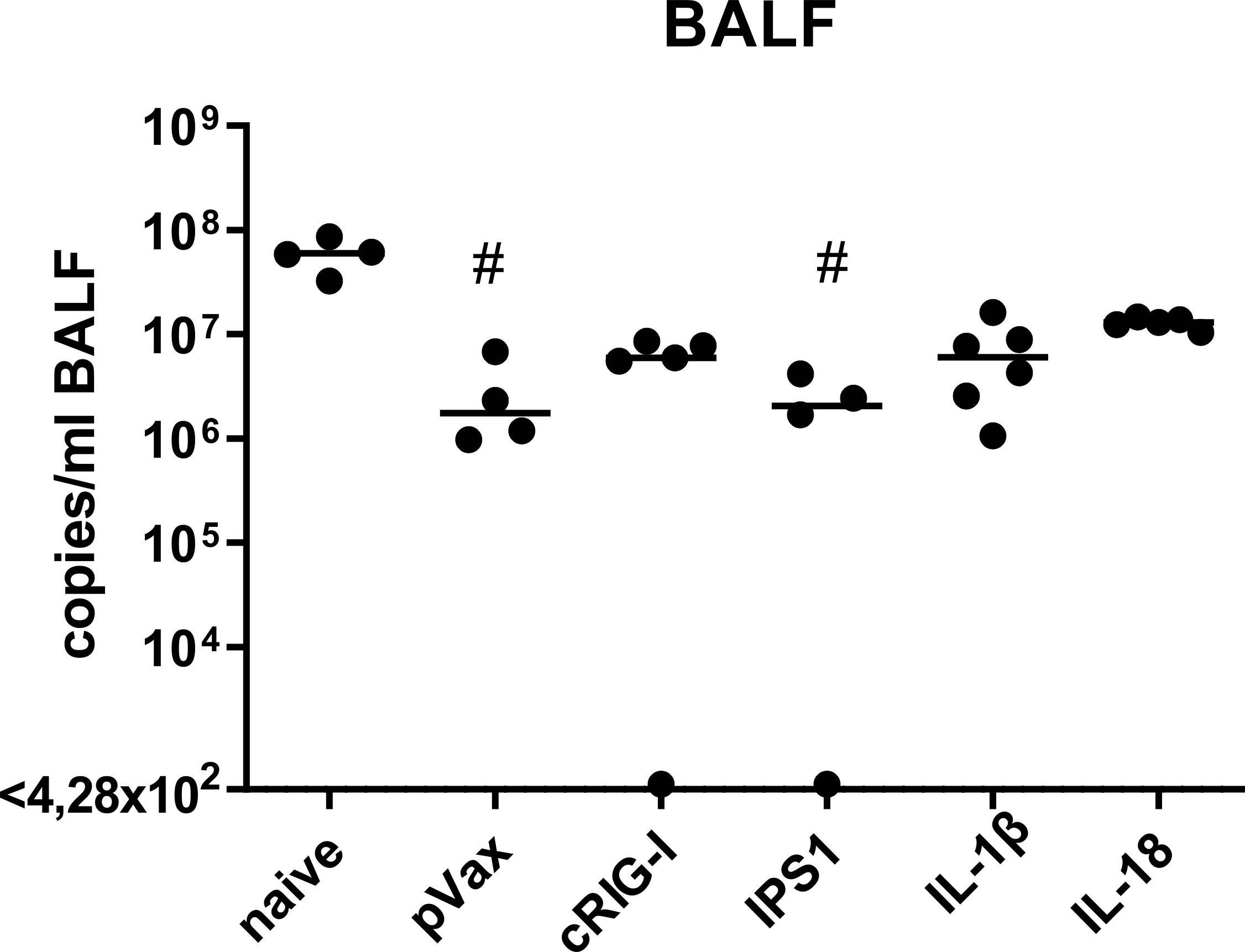


**Fig. S3: qPCR analysis of viral RNA in BALF.**

Supplement: S3 Fig — BALB/c mice were immunized with 5 μg pVax-HA, 5 μg pVax-NP, and 10 μg total adjuvant DNA via intramuscular injection followed by electroporation. 35 days after the immunization, mice were infected with 5000 PFU pH1N1 A/Hamburg/4/2009. Eight days post-infection, mice were euthanized, bronchoalveolar lavages were performed, and virus replication was analysed by qRT-PCR in BALF. The results of the qRT-PCR are displayed as individual data point for each animal and the group median (n = 4–6 animals per group). #, p < 0.05 vs. naive; *, p < 0.05 vs. pVax-empty (Kruskal-Wallis non-parametric one-way ANOVA followed by Dunn's post-test). (DOCX) [file pone.0231138.s003.docx]
